# A study on the 10-year trend of surgeries performed for lumbar disc herniation and comparative analysis of prescribed opioid analgesics and hospitalization duration: 2010–2019 HIRA NPS Data

**DOI:** 10.1186/s12891-024-07167-w

**Published:** 2024-01-13

**Authors:** Sang Yoon Kim, Yu-Cheol Lim, Byung-Kwan Seo, Dongwoo Nam, In-Hyuk Ha, Ye-Seul Lee, Yoon Jae Lee

**Affiliations:** 1 Jaseng Hospital of Korean Medicine, 536 Gangnam-daero, Gangnam-gu, Seoul, 06110 Republic of Korea; 2 Jaseng Spine and Joint Research Institute, Jaseng Medical Foundation, 2F, 540 Gangnam-daero, Gangnam-gu, Seoul, 06110 Republic of Korea; 3 Department of Acupuncture and Moxibustion, Kyung Hee University Korean Medicine Hospital at Gangdong, Seoul, 05278 Republic of Korea; 4 Department of Acupuncture and Moxibustion, Kyung Hee University, Seoul, Republic of Korea

**Keywords:** Opioids, Lumbar surgery, Disc herniation

## Abstract

**Background:**

This study, utilizing the claims data from the Health Insurance Review and Assessment Service of Korea, aimed to examine the 10-year (2010–2019) trends in various types of lumbar spine surgeries performed on patients diagnosed with lumbar herniated intervertebral disc (HIVD), and the current status of opioid prescriptions, as well as the duration of postoperative hospital stays based on the type of surgery performed.

**Method:**

This retrospective cross-sectional study examined patients with one or more national health insurance claims carrying a primary or secondary diagnosis of HIVD (ICD-10 codes: M511, M518, M519) over a 10-year period (2010–2019). From the patients undergoing lumbar spine surgery, we selected those who did not require reoperation within 30 days following the initial lumbar surgery. Our final study sample comprised patients who underwent only one type of surgery.

**Results:**

Among the patients diagnosed with HIVD and subsequently undergoing lumbar surgery between 2010 and 2019, a slight downward trend was observed in those undergoing open discectomy (OD); however, OD persistently accounted for the highest proportion over the 10 years. Percutaneous endoscopic lumbar discectomy (PELD) demonstrated a consistent upward trend from 2016 to 2018. When inspecting trends, we noted a consistent escalation over the decade in the postoperative opioid prescription rates of strong opioids (50.7% in 2010 to 77.8% in 2019) and tramadol (50.9% in 2010 to 76.8% in 2019). Analyzing these trends by surgery type, spinal fusion exhibited a slightly higher rate of opioid prescriptions than other lumbar surgeries. Regarding the length of postoperative hospital stays, patients undergoing PELD recorded the shortest stay (7.04 ± 6.78 days), while spinal fusion necessitated the longest (20.14 ± 12.18 days).

**Conclusion:**

This study analyzed the trends in types of lumbar spine surgeries, opioid analgesic prescriptions, and length of hospital stays over 10 years (2010–2019) among patients with HIVD in Korea. Our data and findings provide valuable evidence that may prove beneficial for clinicians and researchers involved in HIVD-related practices.

**Supplementary Information:**

The online version contains supplementary material available at 10.1186/s12891-024-07167-w.

## Background

Lumbar Herniated Intervertebral Disc (HIVD) is among the most prevalent types of spinal degenerative disorders causing low back pain accompanied by radiculopathy, accounting for approximately two-thirds of spinal pain diagnoses [1–3]. The intervertebral disc is composed of the inner nucleus pulposus (NP) and the outer annulus fibrosus (AF), with HIVD referring to the displacement of the inner disc material NP beyond the disc space limits and the rupture of AF [4]. Two main treatment approaches are surgical and conservative, with surgery often favored for better short- and medium-term outcomes in pain reduction and functional disability improvement [5], due to which an increasing number of patients with HIVD receive surgery [6, 7].

Lumbar spine surgery types include open discectomy (OD), laminectomy, percutaneous endoscopic lumbar discectomy (PELD), spinal fusion, and nucleolysis [8]. In a previous study, OD was the most common procedure followed by endoscopic discectomy [6], known for its effectiveness in pain relief and neurological function restoration. However, it can cause muscle damage, scarring, and spinal instability from nerve retraction [9, 10]. PELD, on the other hand, offers advantages such as shorter operation times, reduced hospital stays, and less hemorrhage [10, 11]. For these reasons, some studies recommend PELD as an alternative to OD [12, 13]. Laminectomy and spinal fusion are also used, but they carry risks of multiple sclerosis, chronic low back pain, and nerve damage [14, 15].

Despite surgical advancements, postoperative pain remains a significant concern, with many patients experiencing moderate pain 6 months after surgery. There is yet no established standard for prescribing analgesics following spinal surgery [16]. Morphine was previously commonly used in clinical practice for pain management [17, 18]; however due to complications like nausea, vomiting, pruritus, and hypotension, efforts have been made to reduce morphine consumption [19, 20]. The type and dose of analgesics vary, with PELD showing promise in short-term preoperative back pain relief [21]. Apart from medical outcomes, surgery also incurs various social costs such as productivity loss and reduced quality of life of patients. Minimizing surgery-induced hospital stays can contribute to patients’ improved psychological well-being and quality of life [22].

Previous study focused on the incidence of OD and PELD [7], but few studies have investigated postoperative medication trends and the duration of the recovery period. This study aims to fill this gap by analyzing 10-year claims data (2010–2019) from the Health Insurance Review and Assessment Service (HIRA) to examine type of lumbar surgery for HIVD, changes in opioid analgesic prescriptions, and postoperative hospital stay durations by surgery type.

## Methods

### Data collection

This study analyzed claims data provided by the Health Insurance Review and Assessment Service (HIRA), specifically the HIRA-National Patient Sample (HIRA-NPS) data spanning from January 2010 to December 2019. The HIRA-NPS is a 2% sample (approximately 1,000,000 individuals) annually selected through sex-stratified (two categories) and age-stratified (16 categories) random sampling from the 98% of the Korean population enrolled in the National Health Insurance (NHI) program. The provided data de-identified any personally identifiable information and include treatment and prescription data based on NHI claims.

### Study design and population

This retrospective cross-sectional study examined patients who made one or more NHI claims with HIVD (ICD-10 code: M511, M518, M519) as the primary or secondary diagnosis over a 10-year period (2010–2019). From this sample, we selected adult patients, aged 20 years or older, who sought care at various medical institutions, including tertiary hospitals, general hospitals, clinics, Korean medicine (KM) hospitals, and KM clinics. Only those with complete data for the study variables were included. Moreover, those who did not require reoperation within 30 days following the initial lumbar surgery were selected. Ultimately, the patients who underwent only one type of surgery among the surgical options considered in this study were finally selected for the study.

### Study outcomes

In this study, we analyzed the baseline characteristics of patients, including age, sex, and payer type (Table 1). The age category was stratified into six groups, each representing a 10-year increment for adults aged 20 years or older. The surgical treatment type (“Surgery type”) for HIVD was classified into OD, laminectomy, PELD, and spinal fusion, and we examined the annual trends in the number and percentage of each surgery type. We categorized prescribed medications during inpatient and outpatient care, including the day of surgery, according to the Anatomical Therapeutic Chemical (ATC) classification system, and investigated the yearly trends of the number and percentage of patients prescribed each medication (Additional Table 1). Among the different medications, opioid analgesics were further divided into strong opioids, weak opioids, and tramadol, and the usage rate for each category was calculated by surgery type. Finally, we also examined trends in hospital stay duration, considered as the recovery period from surgery completion to return to daily routine, by surgery type.

**Table 1:** Baseline characteristics

Category		Total (*n* = 7,741)	
		N	Percentage
Age (years)	20–29	861	11.12
	30–39	1491	19.26
	40–49	1828	23.61
	50–59	1755	22.67
	60–69	1298	16.77
	70–	508	6.56
Gender	Male	4387	56.67
	Female	3354	43.33
Payer type	NHI	7479	96.62
	Medicaid	257	3.32
	Others	5	0.06
Surgery type	OD	6528	84.33
	Laminectomy	748	9.66
	PELD	394	5.09
	Spinal fusion	71	0.92
Surgery medical institution	Tertiary hospital	214	2.76
	General hospital	1180	15.24
	Hospital	6175	79.77
	Clinic	172	2.22
Year of surgery	2010	802	10.36
	2011	747	9.65
	2012	890	11.50
	2013	815	10.53
	2014	794	10.26
	2015	702	9.07
	2016	684	8.84
	2017	733	9.47
	2018	790	10.21
	2019	784	10.13

### Statistical analysis

We employed descriptive statistical analysis for data examination in this study. To present the data for baseline patient characteristics, number of patients by lumbar spine surgery type, number of patients by medication category, and the number of patients prescribed each category of opioid analgesics according to surgery type, we used the applicable number of patients and percentages relative to the total patient number. The yearly trends in lumbar surgery types, use of prescribed medications, and opioids prescription by surgery type were illustrated using graphs. The length of hospital stay by surgery type was presented using mean and standard deviation (SD). Furthermore, subgroup analyses were conducted by sex and age groups (Younger adults below 40 years of age vs. Older adults more than 40 years of age). Calculations and graph creation were performed using the statistical software suite SAS (version 9.4, SAS Institute, Cary, NC, USA).

### Ethical considerations

This study protocol received approval from the public data provision deliberation committee in the HIRA and adhered to relevant guidelines and regulations. The current study was reviewed by the Institutional Review Board of Jaseng Hospital of Korean Medicine, Seoul, Korea (IRB file No. JASENG 2022-12-008), and the need for consent was waived. As the study analyzed publicly available data, no consent was obtained from the individuals whose data was included; all personal information had been de-identified by the NHIS prior to public release. All analyses performed adhered to the principles outlined in the Declaration of Helsinki.

## Results

### Characteristic of patients

**Fig. 1:**
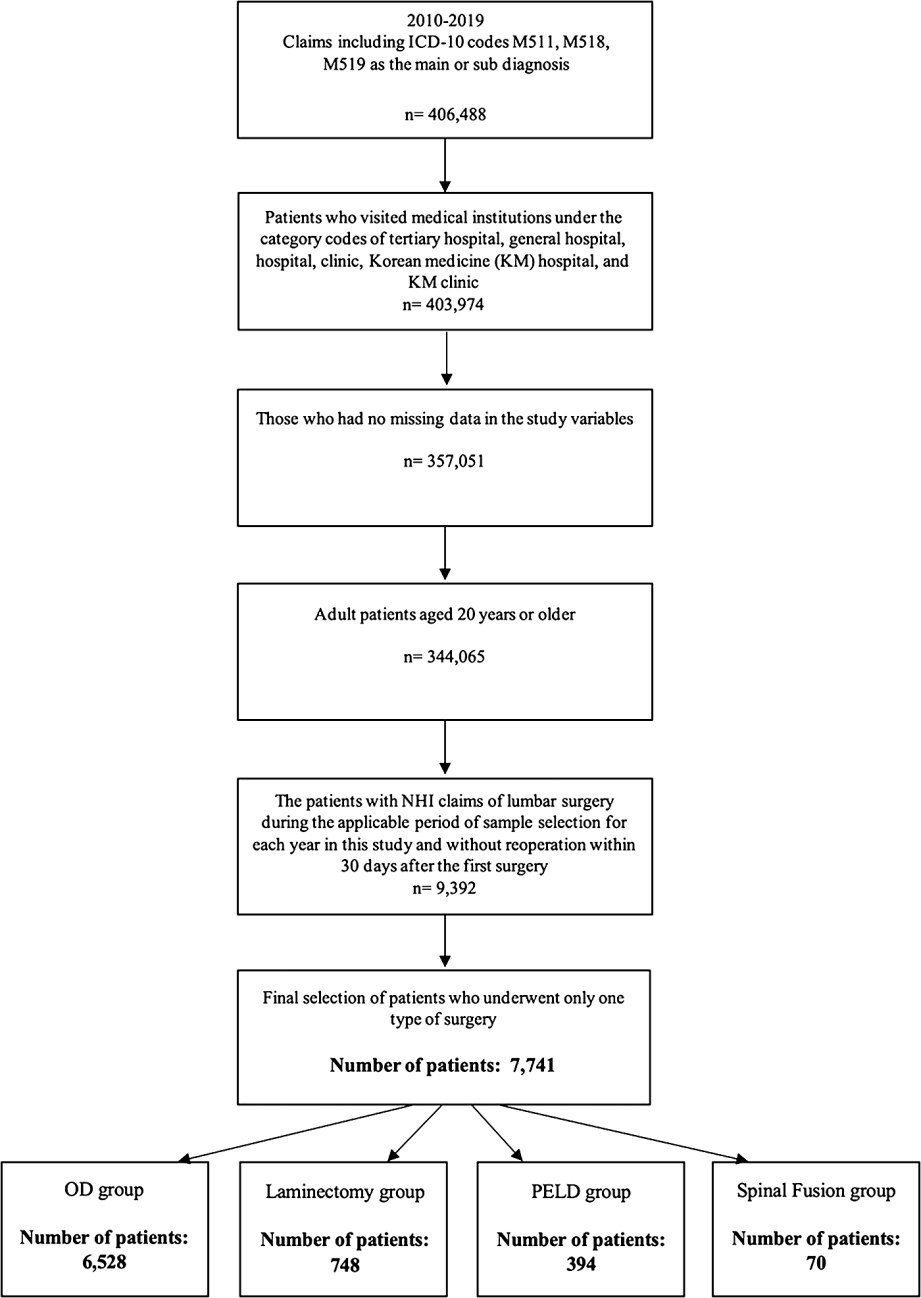
Study patients selection

After the patient selection process, 7741 patients were included in this study (Fig. 1). Regarding the demographic characteristics of the study sample, the age group 40–49 years accounted for highest proportion of patients, followed by the groups 50–59 and 30–39 years. Overall, more male patients were observed compared to female patients, with men making up 56.67% of the total sample. In terms of payer type, almost all patients were covered by the NHI program, accounting for 96.62%. There was no specific noticeable pattens in the yearly trends in the number of patients during the study period. In relation to the surgical treatments received by the patients in this study, OD had the highest ratio, constituting 84.33% of the surgeries. This was followed by laminectomy at 9.66% and PELD at 5.09%. Spinal fusion was rarely performed alone, accounting for only 0.92% over the study period. Regarding the trends in the type of medical institutions, surgery for HIVD was most commonly carried out in hospital-level medical institutions (79.77%), followed by general hospitals, tertiary hospitals, and clinic-level institutions (Table 1).

### Trends in surgical treatment

**Fig. 2:**
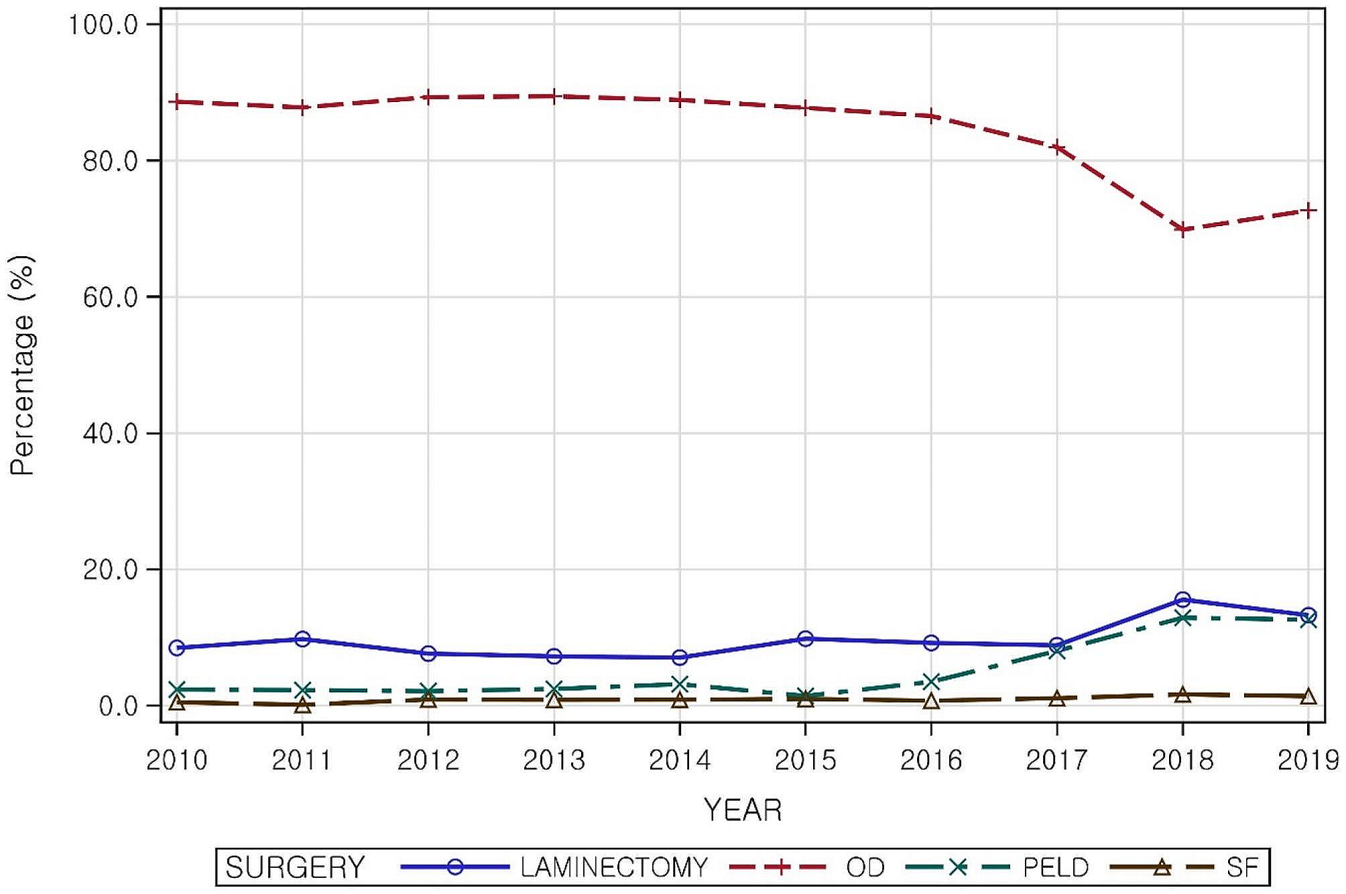
Trends of lumbar surgeries

Figure 2 illustrates a 10-year trend in the proportion of patients who underwent each of the four types of lumbar spine surgery considered in this study. OD was the most common surgery performed throughout the studied decade. However, its proportion started to decline from 2017, reaching its lowest point in 2018 at 69.9%. Laminectomy’s proportion remained stable from 2010 to 2017, but slightly increased to 15.6% in 2018. PELD accounted for a small percentage of approximately 1–3%, but the ratio gradually increased from 3.5% in 2016 to 12.6% in 2019, similar to that of laminectomy. Without much change, spinal fusion remained low at around 0.01% (Additional Table 2).

### Opioid prescription

**Fig. 3:**
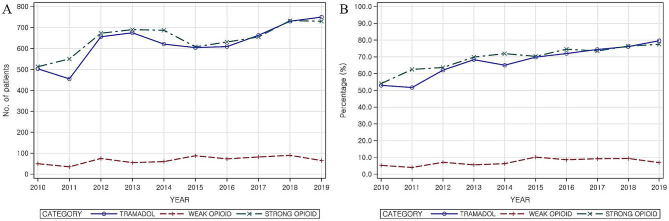
Trends of prescribed opioid analgesics after lumbar surgery. **A**: Number of patients prescribed opioid analgesics (by categories); **B**: Percentage of patients prescribed opioid analgesics (by categories)

The majority of medications prescribed for 1 month (including the day of surgery) for patients who underwent surgery after the diagnosis of HIVD did not show any particular trend over the 10-year period. However, opioid prescriptions saw a steady increase, from about 78% in 2010 to 94% in 2019 (Additional Table 3). Among the types of opioid analgesics, strong opioids and tramadol were predominantly prescribed with a similar trend. The proportion of patients prescribed strong opioids decreased slightly from 50.7% in 2010 to 49.3% in 2011, but subsequently increased to 77.8% in 2019. Tramadol prescriptions also saw an overall increase from 50.9% in 2010 to 76.8% in 2019. In contrast, weak opioids represented less than 1% of total prescriptions over the decade (2010–2019) (Fig. 3).

Considering the trends in opioid prescriptions (including the day of surgery) by surgery type, the prescription rates were similar across laminectomy, OD, and PELD. Among these, strong opioids were prescribed for 64.8% of those who underwent laminectomy, 65.6% for OD, and 61.9% for PELD. Tramadol was prescribed for 66.3% of laminectomy patients, 68% for OD, and 66.5% for PELD. Weak opioids were prescribed for 6.6% of laminectomy patients, 6% for OD, and 8.6% for PELD. However, for spinal fusion, strong opioids were prescribed for 80.3% of patients, weak opioids for 19.7%, and tramadol for 78.9%, showing a comparatively high overall prescription of opioids compared to that for other three surgery types (Table 2). Subgroup analysis did not show differences in the prescription rates by sex (Additional Table 4) or age group (Additional Table 5).

**Table 2:** Prescribed opioid analgesics after lumbar surgery

Category	Laminectomy *n* = 748	OD *n* = 6528	PELD *n* = 394	Spinal fusion *n* = 71
Strong opioids, n (%)	485 (64.84)	4280 (65.56)	244 (61.93)	57 (80.28)
Weak opioids, n (%)	49 (6.55)	390 (5.97)	34 (8.63)	14 (19.72)
Tramadol, n (%)	496 (66.31)	4438 (67.98)	262 (66.50)	56 (78.87)

### Hospital stay

A 10-year trend analysis of the length of hospital stay by surgery type revealed that the average duration of hospital stay for all 6528 surgeries was roughly 13.6 days (SD = 8.95 days). Laminectomy, the second most performed surgery with 748 cases, had an average hospital stay of about 13.0 days (SD = 8.40 days), without a significant difference compared to OD. PELD, a relatively less invasive surgery, had the shortest average hospital stay of 7.04 days (SD = 6.78 days) for a total of 394 surgeries. In contrast, spinal fusion, which involves inserting metallic hardware into the body, had the longest average hospital stay of 20.14 days (SD = 12.18 days) for a total of 71 surgeries (Table 3). Subgroup analysis showed that females stayed slightly longer compared to male in all types of surgery (Additional Table 6), and differences in the duration of hospital stay by age group was not observed (Additional Table 7).

**Table 3:** Duration of hospital stay after lumbar surgery

Inpatient days (day)	Laminectomy	OD	PELD	Spinal fusion
Mean (SD)	13.03 (8.40)	13.61 (8.96)	7.04 (6.78)	20.14 (12.18)
1–5, n (%)	104 (13.90)	603 (9.24)	219 (55.58)	00 (0.00)
6–10	244 (32.62)	2332 (35.72)	109 (27.66)	10 (14.08)
11–15	194 (25.94)	1731 (26.52)	29 (7.36)	18 (25.35)
16–20	95 (12.70)	901 (13.80)	17 (4.31)	22 (30.99)
21–	111 (14.84)	961 (14.72)	20 (5.08)	21 (29.58)

## Discussion

In this study, we used claims data from 2010 to 2019 provided by HIRA to explore the recent 10-year trends in the types of lumbar surgeries performed on patients diagnosed with HIVD. We also examined changes in the prescription of opioid analgesics and the length of postoperative hospital stay according to surgery type. During the 10-year period, OD accounted for the highest proportion of lumbar surgeries, with PELD showing an increasing trend in more recent 4 years. The prescription of strong opioids and tramadol demonstrated an overall upward trend. Spinal fusion had a higher opioid prescription rate compared to other surgery types. Moreover, the postoperative length of hospital stay varied by surgery type, with spinal fusion requiring the longest hospital stay.

In the United States, the number of spinal surgeries for lumbar degenerative disc disease increased 2.4 times between 2000 and 2009. However, the number of lumbar spine surgeries began to decline after 2010. From 2003 to 2013, the number of lumbar discectomies and laminectomies decreased by 19.8% and 26.1%, respectively, while spinal fusion increased by 56.4% [3]. In Korea, from 2003 to 2008, OD was the most common surgery type for patients with lumbar disc herniation, and its proportion increased from 71.21 to 84.12% during this period. On the other hand, PELD decreased from about 16.68–4.57% during the same period. Spinal fusion saw an increase from 3.97 to 6.61% during the same period [23]. In the present study, which is based on the analysis of relatively recent data, OD was the most common type of lumbar surgery for the 10-year study period but began to decrease from 2017.

PELD, showing the most discrepancy between this study and previous studies, has shown the most advancement in terms of technique over the last 10 years as of 2020 [24]. Compared to OD, PELD offers shorter recovery time, faster back pain improvement, and generally fewer complications [25]. However, the learning curve for PELD is steep, and surgeons with insufficient training are likely to face incomplete decompression [25, 26]. Therefore, with technical advancement and skill improvement over time, it is expected that PELD will be more frequently performed among the surgery types, as demonstrated by the present results. However, despite the aforementioned advantages of PELD, there are still conflicting reports that OD yields superior clinical results [27]. In this study, spinal fusion accounted for only a very small proportion of approximately 1% among the surgery types. The considerable difference in proportion of spinal fusion performed in this study and previous studies is believed to be due to the difference in how the surgery is performed in practice; in many cases, spinal fusion is performed along with laminectomy or OD, but in this study, we only included cases where one type of surgery was performed.

According to Jarebi, M. et al., the PELD group had a shorter hospital stay (2.55 ± 1.78 days) compared to the open lumbar microdiscectomy (OLMD) group (3.21 ± 1.26 days; *p* < 0.037) [25]. Moreover, Ahn.S.S et al. reported a shorter hospital stay in the PELD group (7.50 ± 2.63) than in the OLMD group (15.65 ± 4.80, *p* < 0.001). Compared to OLMD, PELD is considered superior in terms of duration of hospital stay and work resumption because PELD has a shorter operation time and causes less intraoperative tissue damage than OLMD [10]. Despite the overall duration of hospital stay in this study being longer compared with that in previous studies due to healthcare service environment differences, the patients who underwent PELD in our study had a relatively shorter hospital stay than those who underwent OD.

In a past study using MarketScan claims data in the US, approximately 23% of patients who underwent lumbar discectomy from 2010 to 2015 used opioids. However, it is noteworthy that in 2010, about 27% of the patients used opioids, but this proportion decreased considerably to about 17% in 2015. Additionally, the use of high-dose opioids decreased from 59% in 2010 to 43% in 2015, and that of very high-dose opioids decreased from 26% in 2010 to 19% in 2015 [28]. Although there is no comparable study on opioid prescription after lumbar surgery in Korea, the total number of opioid prescriptions in Korea displayed a consistent increasing trend, from approximately 17 million cases in 2009 to 27 million cases in 2019. The rate per 1,000 persons of total opioid prescriptions also continued to increase over the last 11 years, from 347.5 in 2009 to 531.3 in 2019 [29]. In this study based on NHI claims data from Korea, the rates of prescribing strong opioids and tramadol during and after surgery increased with similar trend from 50.7% to 50.9% in 2010 to 77.8% and 76.8% in 2019, respectively.

The analysis of opioid prescription rates across different types of surgery revealed that patients undergoing laminectomy, OD, and PELD were prescribed opioids in comparable patterns. In contrast, those receiving spinal fusion exhibited significantly higher prescription rates for strong opioids, weak opioids, and tramadol. Gender-based examination indicated that male patients consistently had higher opioid prescription rates across all categories. Age-wise, younger patients undergoing spinal fusion were notably prescribed more strong opioids, weak opioids, and tramadol. It is important to note that the subset of patients undergoing only spinal fusion was relatively small. Consequently, generalizing these findings requires caution, and additional research is imperative to better understand the influence of spinal fusion surgery types on opioid prescription trends.

The escalating opioid crisis in the United States, which is increasingly gaining global attention [30, 31], underscores the importance of this study. Prior research has indicated an elevated risk of chronic opioid use among post-surgical patients [32, 33]. In this context, our study aimed to evaluate the patterns of opioid prescriptions, including strong and weak opioids as well as tramadol, in patients post-lumbar disc herniation surgery, providing insights into their overall exposure to these medications. However, the cross-sectional nature of the data limits the ability to investigate the relationship between opioid prescription patterns and the duration of hospital stays. Thus, further research is essential to explore this aspect more comprehensively.

In Korea, a more detailed treatment strategy for opioid prescription is needed. Currently, opioid use in Korea is governed by the Standards for Safe Use of Narcotic Analgesics issued by the Ministry of Food and Drug Safety; however, strategies for different treatment types have not been established yet. Moreover, these standards do not include tramadol, which should be addressed in future updates. Conversely, in the United States, where opioid prescriptions kept increasing until the early- and mid-2010s, efforts have been made to reduce opioid consumption through the development of the SOURCE (Simulation of Opioid Use, Response, Consequences, and Effects) model, prescription drug monitoring programs (PDMPs), and various regulations [30, 31]. Similarly, in Korea, where opioid prescriptions have shown an overall increasing trend, there is an urgent need for active discussions on the development of a more specific model for opioid prescription strategy for postoperative pain management.

### Strength & limitation

In the present study, the sample utilized is representative as it used the claims data (HIRA-NPS) sampled from the national population of Korea. The study’s strength lies in examining the recent 10-year trends and healthcare utilization by the type of lumbar surgery performed on patients diagnosed with lumbar HIVD, encompassing patients across all adult age groups over 20 years of age based on age strata provided by the data source. However, this study has several limitations. First, among patients diagnosed with HIVD and those who underwent surgical treatment, all cases that underwent reoperation or required two or more types of lumbar surgeries were excluded based on NHI claims of the applicable year. This could have led to underestimation of the number of patients, prescription of pain medications, or the length of hospital stay. Second, retrieving previous treatment history of the patients was difficult in the study sample as data sources divided by year were used, and follow-up monitoring for long-term use of opioids after surgery was not possible. Third, this study’s data source comprised NHI claims; thus, surgeries not covered by NHI were not included in the analysis. Furthermore, the categorization of certain surgery codes, such as open discectomy, which encompassed both open microdiscectomy and tubular retractor assisted microdiscectomy, was too broad to capture the potential differences that clinicians would have been interested in. Additionally, since there was no information on patient-reported pain scores or quality-of-life, it was not possible to compare the prognosis of surgery in a practical sense. Fourth, only patients who underwent one type of surgery were selected for analysis. However, lumbar fusion is often performed along with OD or laminectomy, but such cases were not considered in this study. Therefore, careful interpretation is required for further generalization of results obtained in this study.

## Conclusion

This comprehensive study, utilizing claims data from HIRA spanning 2010 to 2019, has provided valuable insights into the evolving trends in lumbar surgery types and opioid prescription patterns for patients diagnosed with HIVD in Korea. Our findings indicate that OD remained the most prevalent surgery type throughout the decade, although its frequency began to decline from 2017 onwards. In contrast, PELD showed a notable increase in recent years, likely due to advancements in surgical techniques and its benefits in terms of recovery time and complications. The study highlighted a significant increase in the prescription of strong opioids and tramadol, particularly in spinal fusion cases, which also necessitated longer hospital stays. This trend was more pronounced in younger patients and males, suggesting demographic variations in postoperative pain management strategies. These findings are particularly relevant in the context of the global opioid crisis and the increasing focus on post-surgical pain management. Comparisons with international data, particularly from the United States, reveal some differences in surgical practices and opioid prescription patterns. These differences underscore the need for a more tailored approach to opioid prescription in postoperative pain management in Korea, considering the rising trend in opioid prescriptions. While this study sheds light on important trends in lumbar surgery and opioid prescription in Korea, it also highlights the need for ongoing research and the development of more nuanced strategies for opioid prescription and pain management in post-surgical care. With the global focus on the opioid crisis, such research is crucial for informing healthcare policies and clinical practices that prioritize patient safety and effective pain management.

### Electronic supplementary material

Below is the link to the electronic supplementary material.

Supplementary Material 1

Supplementary Material 2

Supplementary Material 3

Supplementary Material 4

Supplementary Material 5

Supplementary Material 6

Supplementary Material 7

## Data Availability

The datasets analyzed in the current study are available upon authorization by the inquiry committee of research support within HIRA. The Patient Samples are provided in a DVD (text file) format, and a fee is charged for the samples. https://opendata.hira.or.kr/home.do (accessed on 10 July 2023). The contact person for inquiries regarding this data is Ye-Seul Lee.

## Abbreviations

HIVD: Lumbar Herniated Intervertebral Disc
ICD: 10-International Classification of Diseases, 10th Revision
OD: Open Discectomy
PELD: Percutaneous Endoscopic Lumbar Discectomy
SD: Standard Deviation
NP: Nucleus Pulposus
AF: Annulus Fibrosus
HIRA: Health Insurance Review and Assessment Service
NPS: National Patient Sample
NHI: National Health Insurance
OLMD: Open Lumbar Microdiscectomy
SOURCE: Simulation of Opioid Use, Response, Consequences, and Effects
PDMPs: Prescription Drug Monitoring Programs
