# Supplementary material for: A study on the 10-year trend of surgeries performed for lumbar disc herniation and comparative analysis of prescribed opioid analgesics and hospitalization duration: 2010–2019 HIRA NPS Data
Source: BMC Musculoskelet Disord. 2024 Jan 13;25:65. doi: 10.1186/s12891-024-07167-w (PMC10787428; doi:10.1186/s12891-024-07167-w)
Supplement: Supplementary file 1 — Supplementary Material 1 [file 12891_2024_7167_MOESM1_ESM.docx]

| Additional table 2. The number (and percentage) of patients who underwent lumbar surgery from 2010 until 2019 | | | | | | | | | | |
| --- | --- | --- | --- | --- | --- | --- | --- | --- | --- | --- |
| Category | Year, n (%) | | | | | | | | | |
|  | 2010 | 2011 | 2012 | 2013 | 2014 | 2015 | 2016 | 2017 | 2018 | 2019 |
| Laminectomy | 68 (8.48) | 73 (9.77) | 68 (7.64) | 59 (7.24) | 56 (7.05) | 69 (9.83) | 63 (9.21) | 65 (8.87) | 123 (15.57) | 104 (13.27) |
| OD | 711 (88.65) | 656 (87.82) | 795 (89.33) | 729 (89.45) | 706 (88.92) | 616 (87.75) | 592 (86.55) | 601 (81.99) | 552 (69.87) | 570 (72.70) |
| PELD | 19 (2.37) | 17 (2.28) | 19 (2.13) | 20 (2.45) | 25 (3.15) | 10 (1.42) | 24 (3.51) | 59 (8.05) | 102 (12.91) | 99 (12.63) |
| Spinal fusion | 4 (0.50) | 1 (0.13) | 8 (0.90) | 7 (0.86) | 7 (0.88) | 7 (1.00) | 5 (0.73) | 8 (1.09) | 13 (1.65) | 11 (1.40) |
