# Supplementary material for: A study on the 10-year trend of surgeries performed for lumbar disc herniation and comparative analysis of prescribed opioid analgesics and hospitalization duration: 2010–2019 HIRA NPS Data
Source: BMC Musculoskelet Disord. 2024 Jan 13;25:65. doi: 10.1186/s12891-024-07167-w (PMC10787428; doi:10.1186/s12891-024-07167-w)
Supplement: Supplementary file 2 — Supplementary Material 2 [file 12891_2024_7167_MOESM2_ESM.docx]

| Additional table 1. Classification of medication | |
| --- | --- |
| Category | ATC code |
| Opioids | N01(N01AH), N02(N02AA, N02AB, N02AD, N02AE, N02AF, N02AJ, N02AX) |
| Anti-inflammatory  & Analgesics | M01(M01AB, M01AC, M01AE, M01AG, M01AH, M01AX), M02(M02AA), M03(M03AB, M03AC, M03BA, M03BC, M03BX, M03CA), M03(M09AX), N02(N02AA, N02BA N02BE, N02CA, N02CC), N03(N03AE, N03AF, N03AG, N03AX), N04(N04AC, N04BA, N04BC) |
| Anesthetics | N01(N01AB, N01AF, N01AX, N01BB) |
| Gastrointestinal | A01(A01A, A01AD), A02(A02A, A02AA, A02AB, A02AC, A02AD, A02BA, A02BB, A02BC, A02BX, A02X), A03(A03A, A03AA, A03AB, A03AC, A03AE, A03AX, A03BA, A03BB, A03CA, A03F, A03FA), A04(A04AA, A04AD), A05(A05AA, A05AX, A05BA) A06(A06AB, A06AC, A06AD, A06AG), A07(A07AA, A07BC, A07DA, A07EC, A07FA), A09(A09A, A09AA, A09BA), A10(A10BA, A10BB), A12(A12AX), A16(A16AX) |
| Psychometrics | N03(N03AA), N05(N05AA, N05AD, N05AH, N05AX, N05BA, N05BB, N05BE, N05CD, N05CF, N05CM), N06(N06AA, N06AB, N06AX, N06BX, N06DA, N06DX), N07(N07AX, N07BB) |
| Antibiotics  & Antivirals | A07(A07AA), J01(J01AA, J01CA, J01CE, J01CF, J01CR, J01DB, J01DC, J01DD, J01DE, J01DF, J01DH, J01EE, J01FA, J01FF, J01GB, J01MA, J01XA, J01XC, J01XD, J01XX), J02(J02AC), J04(J04AB, J04AC, J04AK), J05(J05AB, J05AF, J05AH, J05AP), P01(P01BA) |
| Others | A01, A06, A10, A11, A12, B01, B02, B03, B05, C01, C02, C03, C04, C05, C07, C08, C09, C10, D01, d02, D03, D05, D06, D07, D08, D11, G01, G03, G04, H01, H02, H03, H05, J06, L01, L02, L03, L04, M01, M04, M05, M09, N07, R01, R02, R03, R05, R06, R07, S01, S02, V03, V04, V07, V08,V09 |
