# Supplementary material for: A study on the 10-year trend of surgeries performed for lumbar disc herniation and comparative analysis of prescribed opioid analgesics and hospitalization duration: 2010–2019 HIRA NPS Data
Source: BMC Musculoskelet Disord. 2024 Jan 13;25:65. doi: 10.1186/s12891-024-07167-w (PMC10787428; doi:10.1186/s12891-024-07167-w)
Supplement: Supplementary file 3 — Supplementary Material 3 [file 12891_2024_7167_MOESM3_ESM.docx]

| Additional table 3. Trends of prescribed medication after lumbar surgery from 2010 until 2019 | | | | | | | | | | |
| --- | --- | --- | --- | --- | --- | --- | --- | --- | --- | --- |
| Category | Year, n (%) | | | | | | | | | |
|  | 2010 | 2011 | 2012 | 2013 | 2014 | 2015 | 2016 | 2017 | 2018 | 2019 |
| ANESTHETIC | 926 (97.58) | 849 (96.48) | 1020 (96.50) | 962 (97.37) | 949 (99.37) | 854 (98.73) | 840 (99.29) | 888 (99.66) | 954 (99.48) | 940 (99.79) |
| ANTIBIOTICS and ANTIVIRALS | 948 (99.89) | 877 (99.66) | 1056 (99.91) | 988 (100.00) | 950 (99.48) | 865 (100.00) | 845 (99.88) | 889 (99.78) | 959 (100.00) | 941 (99.89) |
| ANTIINFLAMMATORY and ANALGESICS | 943 (99.37) | 876 (99.55) | 1048 (99.15) | 985 (99.70) | 951 (99.58) | 861 (99.54) | 841 (99.41) | 884 (99.21) | 953 (99.37) | 937 (99.47) |
| GASTROINTESTINAL | 946 (99.68) | 876 (99.55) | 1055 (99.81) | 985 (99.70) | 951 (99.58) | 864 (99.88) | 843 (99.65) | 888 (99.66) | 957 (99.79) | 938 (99.58) |
| OPIOIDS | 736 (77.56) | 709 (80.57) | 899 (85.05) | 857 (86.74) | 844 (88.38) | 773 (89.36) | 765 (90.43) | 815 (91.47) | 887 (92.49) | 887 (94.16) |
| OTHERS | 949 (100.00) | 880 (100.00) | 1057 (100.00) | 988 (100.00) | 955 (100.00) | 865 (100.00) | 846 (100.00) | 891 (100.00) | 959 (100.00) | 942 (100.00) |
| PSYCHOTOMIMETIC | 587 (61.85) | 534 (60.68) | 623 (58.94) | 549 (55.57) | 552 (57.80) | 527 (60.92) | 470 (55.56) | 507 (56.90) | 571 (59.54) | 501 (53.18) |
