# Supplementary material for: A study on the 10-year trend of surgeries performed for lumbar disc herniation and comparative analysis of prescribed opioid analgesics and hospitalization duration: 2010–2019 HIRA NPS Data
Source: BMC Musculoskelet Disord. 2024 Jan 13;25:65. doi: 10.1186/s12891-024-07167-w (PMC10787428; doi:10.1186/s12891-024-07167-w)
Supplement: Supplementary file 4 — Supplementary Material 4 [file 12891_2024_7167_MOESM4_ESM.docx]

| **Hospital stay after surgery by age group** | | |  |  |  |  |  |  |
| --- | --- | --- | --- | --- | --- | --- | --- | --- |
|  | Laminectomy | | OD | | PELD | | Spinal fusion | |
| Inpatient days | Younger adults | Older adults | Younger adults | Older adults | Younger adults | Older adults | Younger adults | Older adults |
| Mean (SD) | 13.07 (8.90) | 12.96 (7.63) | 13.36 (8.85) | 14.53 (9.28) | 6.73 (6.60) | 8.76 (7.56) | 19.54 (9.87) | 20.88 (14.64) |
| 1-5, n (%) | 67 (15.16) | 37 (12.09) | 499 (9.75) | 104 (7.38) | 190 (56.72) | 29 (49.15) | 00 (0.00) | 00 (0.00) |
| 6-10 | 148 (33.48) | 96 (31.37) | 1902 (37.16) | 430 (30.52) | 95 (28.36) | 14 (23.73) | 7 (17.95) | 3 (9.38) |
| 11-15 | 102 (23.08) | 92 (30.07) | 1324 (25.86) | 407 (28.889) | 23 (6.87) | 6 (10.17) | 8 (20.51) | 10 (31.25) |
| 16-20 | 57 (12.9) | 38 (12.42) | 661 (12.91) | 240 (17.03) | 13 (3.88) | 4 (6.78) | 13 (33.33) | 9 (28.13) |
| 21≤ | 68 (15.38) | 43 (14.05) | 733 (14.32) | 228 (16.18) | 14 (4.18) | 6 (10.17) | 11 (28.21) | 10 (31.25) |
