# Supplementary material for: A study on the 10-year trend of surgeries performed for lumbar disc herniation and comparative analysis of prescribed opioid analgesics and hospitalization duration: 2010–2019 HIRA NPS Data
Source: BMC Musculoskelet Disord. 2024 Jan 13;25:65. doi: 10.1186/s12891-024-07167-w (PMC10787428; doi:10.1186/s12891-024-07167-w)
Supplement: Supplementary file 5 — Supplementary Material 5 [file 12891_2024_7167_MOESM5_ESM.docx]

**Additional Table 4. Prescribed narcotic painkillers after surgery by sex**

| Category | Laminectomy | | | OD | | | PELD | | | Spinal fusion | | |
| --- | --- | --- | --- | --- | --- | --- | --- | --- | --- | --- | --- | --- |
|  | Male (n=376) | Female (n=372) |  | Male (n=3752) | Female (n=2776) |  | Male (n=223) | Female (n=171) |  | Male (n=36) | Female (n=35) |  |
|  | n (%) | | *p*-value | n (%) | | *p*-value | n (%) | | *p*-value | n (%) | | *p*-value |
| Strong opioids | 253 (67.29) | 232 (62.37) | 0.43 | 2445 (65.17) | 1835 (66.10) | 0.21 | 134 (60.09) | 110 (64.33) | 0.76 | 33 (91.67) | 24 (68.57) | 0.89 |
| Weak opioids | 27 (7.18) | 22 (5.91) |  | 205 (5.46) | 185 (6.66) |  | 19 (8.52) | 15 (8.77) |  | 8 (22.22) | 6 (17.14) |  |
| Tramadol | 241 (64.10) | 255 (68.55) |  | 2504 (66.74) | 1934 (69.67) |  | 136 (60.99) | 126 (73.68) |  | 30 (83.33) | 26 (74.29) |  |
