# Supplementary material for: A study on the 10-year trend of surgeries performed for lumbar disc herniation and comparative analysis of prescribed opioid analgesics and hospitalization duration: 2010–2019 HIRA NPS Data
Source: BMC Musculoskelet Disord. 2024 Jan 13;25:65. doi: 10.1186/s12891-024-07167-w (PMC10787428; doi:10.1186/s12891-024-07167-w)
Supplement: Supplementary file 6 — Supplementary Material 6 [file 12891_2024_7167_MOESM6_ESM.docx]

| **Hospital stay after surgery by sex** | |  |  |  |  |  |  |  |
| --- | --- | --- | --- | --- | --- | --- | --- | --- |
|  | Laminectomy | | OD | | PELD | | Spinal fusion | |
| Inpatient days | Male | Female | Male | Female | Male | Female | Male | Female |
| Mean (SD) | 11.82 (8.47) | 14.24 (8.17) | 12.67 (8.34) | 14.88 (9.59) | 5.77 (4.49) | 8.70 (8.66) | 19.61 (14.77) | 20.69 (8.96) |
| 1-5, n (%) | 78 (20.74) | 26 (6.99) | 603 (9.24) | 184 (6.63) | 219 (55.58) | 83 (48.54) | 00 (0.00) | 00 (0.00) |
| 6-10 | 121 (32.18) | 123 (33.06) | 2,332 (35.72) | 901 (32.46) | 109 (27.66) | 48 (28.07) | 10 (14.08) | 2 (5.71) |
| 11-15 | 95 (25.27) | 99 (26.61) | 1,731 (26.52) | 721 (25.97) | 29 (7.36) | 14 (8.19) | 18 (25.35) | 11 (31.43) |
| 16-20 | 43 (11.44) | 52 (13.98) | 901 (13.80) | 455 (16.39) | 17 (4.31) | 10 (5.85) | 22 (30.99) | 7 (20.00) |
| 21≤ | 39 (10.37) | 72 (19.35) | 961 (14.72) | 515 (18.55) | 20 (5.08) | 16 (9.36) | 21 (29.58) | 15 (42.86) |
