# Supplementary material for: A study on the 10-year trend of surgeries performed for lumbar disc herniation and comparative analysis of prescribed opioid analgesics and hospitalization duration: 2010–2019 HIRA NPS Data
Source: BMC Musculoskelet Disord. 2024 Jan 13;25:65. doi: 10.1186/s12891-024-07167-w (PMC10787428; doi:10.1186/s12891-024-07167-w)
Supplement: Supplementary file 7 — Supplementary Material 7 [file 12891_2024_7167_MOESM7_ESM.docx]

**Prescribed narcotic painkillers after surgery by age group**

| Category | Laminectomy | | | OD | | | PELD | | | Spinal fusion | | |
| --- | --- | --- | --- | --- | --- | --- | --- | --- | --- | --- | --- | --- |
|  | Younger adults (n=442) | Older adults (n=306) |  | Younger adults (n=5119) | Older adults (n=1409) |  | Younger adults (n=335) | Older adults (n=59) |  | Younger adults (n=39) | Older adults (n=32) |  |
|  | n (%) | | *p*-value | n (%) | | *p*-value | n (%) | | *p*-value | n (%) | | *p*-value |
| Strong opioids | 284 (64.25) | 201 (65.69) | 0.97 | 3340 (65.25) | 940 (66.71) | 0.21 | 203 (60.60) | 41 (69.49) | 0.83 | 33 (84.62) | 24 (75.00) | 0.54 |
| Weak opioids | 29 (6.56) | 20 (6.54) |  | 293 (5.72) | 97 (6.88) |  | 29 (8.66) | 5 (8.47) |  | 9 (23.08) | 5 (15.63) |  |
| Tramadol | 287 (64.93) | 209 (68.30) |  | 3406 (66.54) | 1032 (73.24) |  | 223 (66.57) | 39 (66.10) |  | 28 (71.79) | 28 (87.50) |  |
